# Effect of autoimmune diseases on incidence and survival in subsequent multiple myeloma

**DOI:** 10.1186/1756-8722-5-59

**Published:** 2012-10-02

**Authors:** Kari Hemminki, Xiangdong Liu, Asta Försti, Jianguang Ji, Jan Sundquist, Kristina Sundquist

**Affiliations:** 1 Division of Molecular Genetic Epidemiology, German Cancer Research Center (DKFZ), Im Neuenheimer Feld 580, Heidelberg, D-69120, Germany; 2 Center for Primary Health Care Research, Lund University, Malmö, 205 02, Sweden; 3 College of Lab Medicine, Hebei North University, Zhangjiakou, 075800, China; 4 Stanford Prevention Research Center, Stanford University School of Medicine, Stanford, CA, 94305-5705, USA

**Keywords:** Autoimmune disease, Multiple myeloma, Incidence, Survival

## Abstract

**Background:**

Patients with many types of autoimmune diseases (AIDs) are at an increased risk of cancer, which may depend on underlying dysregulation of the immune system or treatment. We systematically analyzed myeloma risk and survival in patients diagnosed with 33 different AIDs.

**Methods:**

Data on patients with AIDs were retrieved from the Swedish Hospital Discharge Register and were linked to myeloma diagnoses from the Cancer Registry. Standardized incidence ratios (SIR) and hazard ratios (HRs) were calculated for subsequent myeloma between 1964 and 2008.

**Results:**

Among patients with the 33 AIDs analyzed, 457 cases of myeloma were diagnosed. The overall SIR for myeloma was 1.12 and the overall HR was 0.92 and non-significant. SIRs for myeloma were significantly increased after ankylosing spondylitis (2.02) and systemic sclerosis (2.63). Only the HR for myeloma after rheumatic fever (5.27) was significantly increased. The SIR for myeloma before age 60 years was 1.45; the SIR for myeloma was only increased in the period 1964–1990 (1.31) and not later (1.04). Only the SIR for myeloma after ankylosing spondylitis was increased in the period 1991–2008 (2.09); the HRs for myeloma were increased after polymyositis/dermatomyositis (6.44) and rheumatic fever (4.43) but there were only three deaths of myeloma after these AIDs.

**Conclusions:**

The present data showed an increase in myeloma SIR after two AIDs, ankylosing spondylitis and systemic sclerosis, and in HR after rheumatic fever. The overall myeloma risk after any AID was no longer increased in the latter follow-up period of 1991 through 2008.

## Introduction

Autoimmune (AI) diseases (AIDs) constitute many different diseases with systemic or local manifestations, which jointly affect 5 to 10% of the population in the developed countries [1]. AIDs are characterized by the activation of T cells or B cells, or both, in the absence of an ongoing infection. The normal function of these cells is to recognize specific foreign antigens, but they possess a low level of autoreactivity towards own (self or auto) antigens. In AIDs, autoreactivity is increased and symptoms develop [2]. Many cancers are increased in AID patients for reasons that are not known [3-6]. The immunological disturbances in AIDs may be an important mechanism to cancer formation because cancers typically arise in organs with AI manifestations [3-6]. However, treatment with immunosuppressive therapy may also contribute [7]. Many AIDs are treated with nonsteroidal anti-inflammatory drugs and as these are protective against many cancers, they may help to hide carcinogenic effects of AIDs [8].

Multiple myeloma is an incurable malignant disease of clonal plasma cells, which accumulate in the bone marrow and cause clinical sequelae characterized by the production of monoclonal protein, displacement of normal hematopoiesis, bone marrow neovascularization, formation of osteolytic bone lesions, hypercalcemia and renal failure [9]. Myeloma arises from B-cells with somatic hypermutations in the variable region immunoglobin genes [10]. Hypermutations occur in germinal centers and thus myeloma development is thought to be related to post-germinal center immune stimulation. Chronic antigenic stimulation has long been suggested to drive myeloma development [11]. Already in 1987, a personal history of rheumatic fever and urinary tract infections was associated with myeloma risk [12]. Myeloma risks have also been associated with pernicious anemia and polymyalgia rheumatic in previous Swedish studies [3,13]. In US veterans, myeloma risk was associated with polymyositis/dermatomyositis, systemic sclerosis, autoimmune hemolytic anemia, pernicious anemia and ankylosing spondylitis [4]. The data on rheumatoid arthritis has been controversial and data on Crohn disease and sarcoidosis have been negative [3,14,15]. Whether AID influences myeloma survival has deserved less attention and the studies on survival after rheumatoid arthritis, Crohn disease, ulcerative colitis and psoriasis have shown no effect [16-18].

In the present study we examined risks of myeloma (standardized incidence ratio, SIR) and survival in myeloma (hazard ratio, HR) systematically in patients, who had been hospitalized for any of 33 AIDs, covering the total Swedish population. As myeloma treatment has changed over the years with improvements in survival, we analyzed also periodic effects [19].

## Material and methods

The research dataset used in the present study is a subset of the national dataset maintained at the Center for Primary Health Care Research, Lund University, Malmö, Sweden, and has been used previously [6]. AID patients were identified from the Swedish Hospital Discharge Register, which contains data on all hospital discharges (dates of hospitalization and diagnoses) for some regions of Sweden since 1964 and for the whole of Sweden since 1986. The International Classification of Diseases codes used have been described previously [20,21]. A total of 33 AIDS were covered. However, for 12 AIDs (Addison’s disease, amyotrophic lateral sclerosis, autoimmune hemolytic anemia, celiac disease, chorea minor, type 1 diabetes, discoid lupus, localized scleroderma, lupoid hepatitis, polyarteritis nodosa, primary biliary cirrhosis and Reiter’s disease) there were fewer than four cases of myeloma. These diseases were not significantly associated with myeloma (as assessed by SIR and HR) and data are not shown for them. The linkages were performed by means of the individual national identification number that is assigned to each person in Sweden for their lifetime. This number was replaced by a serial number for each person in order to provide anonymity.

SIRs were calculated as the ratio of observed to expected number of myelomas. Expected numbers were calculated for anyone not hospitalized for AID. The expected numbers were calculated as age (5-year groups), sex, period (5-year groups), region and socioeconomic status-specific standard incidence rates. Additional adjustments were made for hospitalization for obesity using codes ICD7 = 287.00, 287.09; ICD8 = 277.99; ICD9 = 278A; ICD-10= E65-E68. A total of 30,020 individuals had been hospitalized for obesity. Similar adjustments were made for smoking using hospitalization for chronic obstructive pulmonary disease as a surrogate with 260,243 individuals affected (codes: ICD7 = 500–502; ICD8 = 490–493; ICD9 =490-496; ICD-10= J40-J49), and alcohol using alcoholisms as surrogate (codes: ICD9 =303; ICD-10=F10.1-F10.9) with 181,862 individuals affected. Confidence intervals (95%CI) were calculated assuming a Poisson distribution.

Person-years of follow-up were calculated from date of discharge with the first main diagnosis of AID until death, emigration, or closing date, December 31, 2008. The Cox regression analyses were used to estimate HRs, which could be interpreted as mortality rate ratio. Adjustments were done as above. The analyses were for cause-specific deaths and deaths from other causes were censored. The proportional hazard assumption for the covariates was tested by Schoenfeld residuals and by plotting the log of the negative log of the survival function versus the log of time.

The study was approved by the regional ethical review board at Lund.

## Results

The numbers of AID patients are shown in Table 1 after the name of the disease and the first column shows the numbers of person-years at risk. Of the 33 AIDs analyzed, 21 had more than 3 cases of myelomas and these are shown in the Tables. For myeloma after any AID, including the ones not shown, the SIR was increased to 1.12, while the HR was 0.92 (not significant, Table 1). SIRs were significantly increased after ankylosing spondylitis (2.02) and systemic sclerosis (2.63). No SIR was significantly decreased. Only the HR after rheumatic fever (5.27) was significantly increased.

**Table 1:** SIRs and HRs for myeloma after a specified AI disease

**Autoimmune disease (case numbers)**	**Pyrs**	**O**	**SIR**	**95%CI**	**Deaths**	**HR**	**95%CI**
**Ankylosing spondylitis (6646)**	112824	16	**2.02**	**1.15-3.28**	**4**	**0.60**	**0.22-1.59**
Behcet disease (3874)	63182	6	1.48	0.53-3.23	3	0.64	0.21-1.98
Chronic rheumatic heart disease (21027)	166242	23	0.92	0.58-1.39	9	0.93	0.48-1.79
Crohn disease (25677)	392264	15	0.82	0.46-1.36	9	1.20	0.62-2.31
Graves/hyperthyroidism (42020)	541104	52	1.03	0.77-1.35	29	0.88	0.61-1.27
Hashimoto/hypothyroidism (13160)	120705	15	1.14	0.64-1.89	4	0.40	0.15-1.06
Immune thrombocytopenic purpura (4324)	44220	4	2.13	0.55-5.51	2	0.57	0.14-2.30
Multiple sclerosis (14616)	185014	13	0.99	0.53-1.70	8	1.25	0.62-2.49
Myasthenia gravis (3044)	32648	4	1.35	0.35-3.49	3	1.85	0.60-5.74
Pernicious anemia (11590)	67096	13	1.01	0.54-1.74	12	1.15	0.65-2.03
Polymyalgia rheumatica (27534)	253046	43	1.20	0.87-1.62	27	1.05	0.72-1.53
Polymyositis/dermatomyositis (2465)	24041	4	1.80	0.47-4.65	3	1.41	0.45-4.38
Psoriasis (19777)	275971	23	0.98	0.62-1.47	11	0.75	0.41-1.35
Rheumatic fever (4306)	77388	8	1.34	0.57-2.65	6	**5.27**	2.37-11.75
Rheumatoid arthritis (72309)	731954	81	0.88	0.70-1.09	45	0.93	0.69-1.24
Sarcoidosis (11571)	177765	19	1.30	0.78-2.03	11	1.05	0.58-1.90
Sjögren syndrome (1957)	16700	4	2.10	0.55-5.42	1	0.30	0.04-2.13
Systemic lupus erythematosus (7624)	86627	11	1.71	0.85-3.07	5	1.10	0.46-2.63
Systemic sclerosis (7169)	89997	19	**2.63**	1.58-4.11	13	0.92	0.53-1.60
Ulcerative colitis (33493)	486704	38	1.36	0.96-1.87	21	0.96	0.62-1.47
Wegener granulomatosis (15833)	115746	26	1.17	0.76-1.72	6	0.77	0.47-1.26
All (402462)	4777665	457	**1.12**	1.02-1.23	251	0.92	0.81-1.04

Bold type indicates that the 95% CI does not include 1.00.

Abbreviations: Pyrs, person-years; O, observed; SIR, standardized incidence ratio; CI, confidence interval; HR, hazard ratio.

The data were analyzed according to the age at myeloma diagnosis (Table 2). Note that the number of patients followed for myeloma was the same as in Table 2. The overall risk of myeloma was increased only for those diagnosed before age 60 years (SIR 1.45); the significant SIRs were noted for ankylosing spondylitis (3.31), chronic rheumatic heart disease (2.79) and systemic lupus erythematosus (5.19). The HRs in young patients were increased after Graves/hyperthyroidism (2.48), rheumatic fever (21.17, but only one death) and systemic sclerosis (5.51). In myeloma patients diagnosed at age 60+ years, only the risk for systemic sclerosis was increased (2.59). HRs were increased after polymyositis/dermatomyositis (5.40) and rheumatic fever (4.60).

**Table 2:** SIRs and HRs for myeloma after a specified AI disease by age at cancer diagnosis

		**0<Age<60**						**Age60**					
**Autoimmune disease**	**Pyrs**	**O**	**SIR**	**95%CI**	**Deaths**	**HR**	**95%CI**	**O**	**SIR**	**95%CI**	**Deaths**	**HR**	**95%CI**
Ankylosing spondylitis	112824	8	**3.31**	1.41-6.55	3	1.00	0.32-3.11	8	1.45	0.62-2.87	1	0.29	0.04-2.01
Behcet disease	63182	2	1.99	0.19-7.32	0			4	1.31	0.34-3.38	3	1.05	0.34-3.24
Chronic rheumatic heart disease	166242	6	**2.79**	1.00-6.11	4	1.25	0.47-3.33	17	0.75	0.43-1.20	5	0.82	0.34-1.96
Crohn disease	392264	5	0.89	0.28-2.09	3	1.47	0.47-4.57	10	0.79	0.38-1.46	6	1.12	0.50-2.50
Graves/hyperthyroidism	541104	7	0.98	0.39-2.04	5	**2.48**	1.03-5.98	45	1.04	0.76-1.39	24	0.80	0.53-1.19
Hashimoto/hypothyroidism	120705	1	0.85	0.00 -4.87	0			14	1.17	0.64-1.97	4	0.44	0.16-1.16
Immune thrombocytopenic purpura	44220	0						4	2.51	0.65-6.50	2	0.57	0.14-2.27
Multiple sclerosis	185014	3	0.86	0.16-2.56	0			10	1.04	0.49-1.92	8	1.84	0.92-3.68
Myasthenia gravis	32648	1	2.60	0.00-14.91	1	2.41	0.34-17.14	3	1.16	0.22-3.45	2	1.55	0.39-6.18
Pernicious anemia	67096	1	2.38	0.00-13.63	1	0.49	3.06-3.48	12	0.97	0.50-1.70	11	1.30	0.72-2.35
Polymyalgia rheumatica	253046	5	1.93	0.61-4.55	3	0.94	0.30-2.92	38	1.14	0.81-1.57	24	1.05	0.70-1.56
Polymyositis/dermatomyositis	24041	1	3.19	0.00-18.26	0			3	1.57	0.30-4.65	3	**5.40**	1.75-16.65
Psoriasis	27591	5	1.19	0.38-2.80	1	0.43	0.06-3.06	18	0.94-	0.55-1.48	10	0.80	0.43-1.49
Rheumatic fever	77388	1	0.76	0.00-4.35	1	**21.17**	2.96-151.59	7	1.50	0.60-3.11	5	**4.60**	1.91-11.06
Rheumatoid arthritis	731954	8	0.90	0.39-1.79	2	0.45	0.11-1.89	73	0.88	0.69-1.10	43	0.96	0.71-1.29
Sarcoidosis	177765	4	1.26	0.33-3.27	2	1.19	0.30-4.78	15	1.31	0.73-2.16	9	1.07	0.55-2.05
Sjögren syndrome	16700	1	4.58	0.00-26.27	0			3	1.78	0.33-5.26	1	0.34	0.05-2.38
Systemic lupus erythematosus	86627	6	**5.19**	1.87-11.36	3	1.42	0.46-4.41	5	0.95	0.30-2.23	2	0.86	0.22-3.45
Systemic sclerosis	89997	3	2.87	0.54-8.49	3	**5.51**	1.75-17.29	16	**2.59**	1.47-4.2	10	0.80	0.43-1.49
Ulcerative colitis	486704	11	1.50	0.75-2.70	6	0.85	0.38-1.89	27	1.31	0.86-1.90	15	1.05	0.63-1.74
Wegener granulomatosis	115746	2	3.91	0.37-14.36	2	1.91	0.48-7.68	24	1.11	0.71-1.65	14	0.71	0.42-1.20
All	4777665	84	**1.45**	1.16-1.80	40	0.96	0.70-1.32	373	1.07	0.96-1.18	211	0.92	0.80-1.06

Bold type indicates that the 95% CI does not include 1.00.

Abbreviations: Pyrs, person-years; O, observed; SIR, standardized incidence ratio; CI, confidence interval; HR, hazard ratio.

In Table 3 the data were analyzed in two periods, 1964 to 1990 and 1991 to 2008; the patient numbers are shown in the parentheses. The overall SIR was only increased when myeloma was diagnosed in years 1964 to 1990 (1.31), systemic sclerosis showing the only significant association (3.60). The overall HR was not changed for this period, but increased HRs for myeloma were noted after multiple sclerosis (4.02), rheumatic fever (5.06) and systemic lupus erythematosus (15.48). In period 1991–2008, the overall SIR was not increased and myeloma was increased only after ankylosing spondylitis (2.09). HRs for myeloma were increased in polymyositis/dermatomyositis (6.44) and rheumatic fever (4.43) patients.

**Table 3:** SIRs and HRs for myeloma after a specified AI disease by diagnostic period of cancer

**Autoimmune disease(case numbers in the two periods)**	**1964-1990**							**1991-2008**						
	**Pyrs**	**O**	**SIR**	**95%CI**	**Deaths**	**HR**	**95%CI**	**Pyrs**	**O**	**SIR**	**95%CI**	**Deaths**	**HR**	**95%CI**
Ankylosing spondylitis (4692/1954)	39322	3	1.73	0.33-5.12	1	3.60	0.50-25.72	73502	13	**2.09**	1.11-3.59	2	0.40	0.10-1.59
Behcet disease (3635/239)	28513	3	2.14	0.40-6.35	3	1.38	0.45-4.29	34669	3	1.12	0.21-3.33	0		
Chronic rheumatic heart disease (17015/4012)	85701	13	1.16	0.62-1.99	6	1.55	0.70-3.46	80541	10	0.73	0.35-1.35	2	0.43	0.11-1.73
Crohn disease (12733/12944)	110614	3	1.01	0.19-3.00	1	1.19	0.17 -8.43	281650	12	0.78	0.40-1.37	6	1.22	0.55-2.71
Graves/hyperthyroidism (26633/15387)	203693	22	1.32	0.82-2.00	8	0.61	0.31-1.23	337411	30	0.89	0.60-1.27	15	1.17	0.71-1.95
Hashimoto/hypothyroidism (8375/4785)	43501	8	1.88	0.80-3.72	3	1.16	0.37-3.59	77204	7	0.79	0.31-1.64	0		
Immune thrombocytopenic purpura (965/3359)	8640	1	4.44	0.00-25.4	1	0.53	0.07-3.73	355800	3	1.81	0.34-5.37	1	0.61	0.09-4.35
Multiple sclerosis (8782/5834)	67706	4	1.03	0.27-2.67	2	**4.02**	1.01-16.01	117308	9	0.97	0.44-1.86	5	1.14	0.47-2.73
Myasthenia gravis (1551/1493)	10430	2	2.91	0.27-10.7	0			22218	2	0.88	0.08-3.23	2	1.92	0.48-7.67
Pernicious anemia (9687/1903)	32746	5	0.83	0.26-1.96	3	0.72	0.23-2.22	34350	8	1.17	0.50-2.32	7	1.37	0.66-2.88
Polymyalgia rheumatica (13278/14256)	72444	10	1.59	0.76-2.94	5	1.35	-0.563.24	180602	33	1.12	0.77-1.57	18	0.92	0.581-1.46
Polymyositis/dermatomyositis (1295/1170)	8310	1	1.57	0.00-9.00	0			15731	3	1.89	0.36-5.59	3	**6.44**	2.08-20.0
Psoriasis (14068/5709)	112458	11	1.51	0.75-2.71	5	0.78	0.32-1.88	163513	12	0.74	0.38-1.30	2	0.31	0.08-1.24
Rheumatic fever (3888/418)	34274	4	2.05	0.53-5.30	2	**5.06**	1.26-20.28	43114	4	0.99	0.26-2.57	3	**4.43**	1.43-13.7
Rheumatoid arthritis (49139/23170)	304697	35	1.02	0.71-1.41	16	0.81	0.49-1.32	427257	46	0.80	0.58-06	25	1.19	0.80-1.77
Sarcoidosis (7856/3715)	69124	9	2.17	0.98-4.14	6	1.31	0.59-2.93	108641	10	0.95	0.45-1.76	3	0.75	0.24-2.32
Sjögren syndrome (716/1241)	3386	1	3.10	0.00-17.7	1	0.87	0.12-6.15	13314	3	1.89	0.36-5.60	0		
Systemic lupus erythematosus (4717/2907)	31854	4	1.93	0.50-4.99	4	**15.48**	5.79-41.42	54773	7	1.60	0.64-3.32	1	0.25	0.04-1.80
Systemic sclerosis (5795/1374)	36494	10	**3.60**	1.71-6.64	7	0.91	0.43-1.93	53503	9	2.02	0.92-3.85	5	1.35	0.56-3.25
Ulcerative colitis (17071/16422)	136018	5	0.95	0.30-2.23	1	0.27	0.04-1.95	350686	33	1.45	1.00-2.04	19	1.46	0.93-2.29
Wegener granulomatosis (14233/1600)	61898	14	1.20	0.65-2.02	3	0.32	0.10-1.00	53848	12	1.14	0.59-2.00	10	1.22	0.66-2.28
All (253449/149013)1696388	1696388	171	**1.31**	1.12-1.52	78	0.88	0.70-1.10	3081277	286	1.04	0.92-1.16	137	0.99	0.99-1.17

Bold type indicates that the 95% CI does not include 1.00.

Abbreviations: Pyrs, person-years; O, observed; SIR, standardized incidence ratio; CI, confidence interval; HR, hazard ratio.

## Discussion

Myeloma remains a fatal disease even though the relative survival in Sweden has increased from 19% in 1964 to 39% in 2003 [22]. The improvements have been ascribed to more effective treatment modalities, which include melphalan-prednisone covering the present study period and interferon alpha since the late 1970s [19]. High-dose melphalan therapy and autologous stem-cell transplantation were introduced in Sweden in the late 1980 and thalidomide began to be used about a decade later [19]. Thus, in the latter part of our follow-up period (1991–2008), many therapeutic innovations were in use, and the overall myeloma risk and survival were identical in AID patients and the population not hospitalized for AIDs. The risk of myeloma remained elevated for ankylosing spondylitis patients and was still of borderline significance for systemic sclerosis patients, although the SIR had decreased compared to the earlier part of the follow-up period (1964–1990). Risk of myeloma before age 60 years was increased in chronic rheumatic heart disease and systemic lupus patients. Ankylosing spondylitis and systemic sclerosis were associated with myeloma in the earlier US study on male veterans [4]. In that study, myeloma risk was associated with a few other AIDs that were not associated with myeloma risk in our study, probably partly for statistical reasons: there were 4,641 cases of myeloma in the US study compared with 457 in the present study. Another reason may be the gender, as only males were included in the US study.

We have been unable to find previous publications documenting adverse (or beneficial) effects of AID for myeloma survival. The reasons for poorer survival in AID patients could be the weak overall physical condition or non-tolerance of therapy, both of which have been described for various cancers [23-25]. In the Swedish study comparing periodic effects on myeloma survival, the most favorable trends were seen for the young patients who also received the most aggressive therapies and were candidates for bone marrow transplantation [19]. The present data showed no overall influence of AID on survival and the null results remained when myeloma diagnostic age groups or periods were considered. However, among individual AIDs, poor survival was observed for rheumatic fever, irrespective of diagnostic age or period. Interestingly, rheumatic fever has previously been shown as a risk factor for myeloma which, however, was not evident in the present study [12]. Rheumatic fever is caused by streptococcal infection usually in childhood. The resulting carditis may develop to congestive heart diseases and this was the likely co-morbidity affecting survival; most of the affected rheumatoid fever patients were 60+ years in the present study. Other AIDs that appeared to worsen survival in myeloma were polymyositis/dermatomyositis (only age 60+ and period 1991 to 2008) and multiple sclerosis and systemic lupus erythematosus (only period 1964 to 1990), all quite devastating AIDs and likely to act as co-morbidities.

The present paper is a continuation in a series of studies on cancers following AIDs, including so far digestive tract, lung cancers and skin cancers [6,26-28]. Although there is no general pattern of certain AIDs leading to increases of all or most cancers, systemic sclerosis, leading to the highest SIR of myeloma, causes also significant increases in esophageal cancer, lung adenocarcinoma and squamous cell carcinoma and skin squamous cell carcinoma. Changes in survival (HR) are not as common as changes in SIR, even in cancers previously studied, at least in part because case numbers are less. We have not previously observed increases in HR of cancer after rheumatic fever.

In conclusion, the present data showed an increase in myeloma SIR after two AIDs (ankylosing spondylitis and systemic sclerosis) and HR after rheumatic fever. The overall myeloma risk after any AID was no longer increased in the latter follow-up period of 1991 through 2008.

## Competing interests

The authors’ declare that they have no competing interests.

## Authors’ contributions

(1) the conception and design of the study, or acquisition of data, or analysis and interpretation of data: all authors, (2) drafting the article or revising it critically for important intellectual content: KH, AF, KS, (3) final approval of the version to be submitted: all authors. All authors read and approved the final manuscript.
